# Genome-Wide Association Study Reveals Multiple Loci Associated with Primary Tooth Development during Infancy

**DOI:** 10.1371/journal.pgen.1000856

**Published:** 2010-02-26

**Authors:** Demetris Pillas, Clive J. Hoggart, David M. Evans, Paul F. O'Reilly, Kirsi Sipilä, Raija Lähdesmäki, Iona Y. Millwood, Marika Kaakinen, Gopalakrishnan Netuveli, David Blane, Pimphen Charoen, Ulla Sovio, Anneli Pouta, Nelson Freimer, Anna-Liisa Hartikainen, Jaana Laitinen, Sarianna Vaara, Beate Glaser, Peter Crawford, Nicholas J. Timpson, Susan M. Ring, Guohong Deng, Weihua Zhang, Mark I. McCarthy, Panos Deloukas, Leena Peltonen, Paul Elliott, Lachlan J. M. Coin, George Davey Smith, Marjo-Riitta Jarvelin

**Affiliations:** 1 Department of Epidemiology and Public Health, Imperial College London, London, United Kingdom; 2 Economic and Social Research Council International Centre for Life Course Studies in Society and Health, London, United Kingdom; 3 Medical Research Council Centre of Epidemiology for Child Health, University College London Institute of Child Health, London, United Kingdom; 4 Medical Research Council Centre for Causal Analyses in Translational Epidemiology, Department of Social Medicine, University of Bristol, Bristol, United Kingdom; 5 Department of Prosthetic Dentistry and Stomatognathic Physiology, Institute of Dentistry, University of Oulu, Oulu, Finland; 6 Oral and Maxillofacial Department, Oulu University Hospital, Oulu, Finland; 7 Department of Oral Development and Orthodontics, Institute of Dentistry, University of Oulu, Oulu, Finland; 8 Clinical Trial Service Unit and Epidemiological Studies Unit (CTSU), University of Oxford, Oxford, United Kingdom; 9 Institute of Health Sciences, University of Oulu, Oulu, Finland; 10 Biocenter Oulu, University of Oulu, Oulu, Finland; 11 Department of Primary Care and Social Medicine, Faculty of Medicine, Imperial College London, United Kingdom; 12 Department of Tropical Hygiene, Faculty of Tropical Medicine, Mahidol University, Bangkok, Thailand; 13 Department of Lifecourse and Services, National Institute of Health and Welfare, Oulu, Finland; 14 Center for Neurobehavioral Genetics, University of California Los Angeles, Los Angeles, California, United States of America; 15 The Jane and Terry Semel Institute for Neuroscience and Human Behavior, Los Angeles, California, United States of America; 16 Department of Psychiatry, University of California Los Angeles, Los Angeles, California, United States of America; 17 Department of Clinical Sciences/Obstetrics and Gynecology, University of Oulu, Oulu, Finland; 18 Finnish Institute of Occupational Health, Oulu, Finland; 19 Department of Oral and Dental Science, University of Bristol, Bristol, United Kingdom; 20 Avon Longitudinal Study of Parents and Children (ALSPAC), University of Bristol, Bristol, United Kingdom; 21 Department of Gastroenterology and Hepatology, Imperial College London, London, United Kingdom; 22 Oxford Centre for Diabetes, Endocrinology and Metabolism (OCDEM), University of Oxford, Oxford, United Kingdom; 23 Wellcome Trust Centre for Human Genetics, University of Oxford, Roosevelt Drive, Headington, Oxford, United Kingdom; 24 Wellcome Trust Sanger Institute, Welcome Trust Genome Campus, Hinxton, Cambridge, United Kingdom; 25 Institute of Molecular Medicine Finland FIMM, Nordic EMBL Partnership for Molecular Medicine, Helsinki, Finland; 26 Department of Medical Genetics, University of Helsinki, Helsinki, Finland; 27 National Institute for Health and Welfare, Public Health Genomics Unit, Helsinki, Finland; 28 Medical Research Council–Health Protection Agency Centre in Environment and Health, London, United Kingdom; Georgia Institute of Technology, United States of America

## Abstract

Tooth development is a highly heritable process which relates to other growth and developmental processes, and which interacts with the development of the entire craniofacial complex. Abnormalities of tooth development are common, with tooth agenesis being the most common developmental anomaly in humans. We performed a genome-wide association study of time to first tooth eruption and number of teeth at one year in 4,564 individuals from the 1966 Northern Finland Birth Cohort (NFBC1966) and 1,518 individuals from the Avon Longitudinal Study of Parents and Children (ALSPAC). We identified 5 loci at *P*<5×10^−8^, and 5 with suggestive association (*P*<5×10^−6^). The loci included several genes with links to tooth and other organ development (*KCNJ2*, *EDA*, *HOXB2*, *RAD51L1*, *IGF2BP1*, *HMGA2*, *MSRB3*). Genes at four of the identified loci are implicated in the development of cancer. A variant within the *HOXB* gene cluster associated with occlusion defects requiring orthodontic treatment by age 31 years.

## Author Summary

Genome-wide association studies have been used to identify genetic variants conferring susceptibility to diseases, intermediate phenotypes, and physiological traits such as height, hair color, and age at menarche. Here we analyze the NFBC1966 and ALSPAC birth cohorts to investigate the genetic determinants of a key developmental process: primary tooth development. The prospective nature of our studies allows us to exploit accurate measurements of age at first tooth eruption and number of teeth at one year, and also provides the opportunity to assess whether genetic variants affecting these traits are associated with dental problems later in the life course. Of the genes that we find to be associated with primary tooth development, several have established roles in tooth development and growth, and almost half have proposed links with the development of cancer. We find that one of the variants is also associated with occlusion defects requiring orthodontic treatment later in life. Our findings should provide a strong foundation for the study of the genetic architecture of tooth development, which as well as its relevance to medicine and dentistry, may have implications in evolutionary biology since teeth represent important markers of evolution.

## Introduction

Heritability of primary tooth emergence is estimated to be over 70% [1]. Abnormalities in tooth development are common with tooth agenesis alone affecting up to 10% of the population, ranking it as the most common developmental anomaly in humans [2]. Such abnormalities contribute to a variety of challenging and expensive orthodontic, prosthetic and surgical treatments and account for approximately 6% of all dental health care attendances [3]. Many genes implicated in primary dentition have regulatory functions important to several developmental processes in the embryo [4], and the developing tooth is a useful model for the study of organogenesis [5]. However, despite substantial research into tooth development in mice and human malformation syndromes [5], the genetic determinants of the normal variation in human tooth development have not been established.

To identify genetic loci regulating primary dentition we performed a general population based genome-wide association (GWA) study of tooth development in infancy among individuals from the 1966 Northern Finland Birth Cohort (NFBC1966) and the Avon Longitudinal Study of Parents and Children (ALSPAC). Specifically, we tested for associations with time to first tooth eruption and number of teeth by one year of age. These phenotypes are relevant to later tooth development because teeth largely acquire their final form at a very early age [6]. The availability of longitudinal birth cohort data allowed us to investigate life-course associations with dental occlusion defects.

## Results

**Figure 1:**
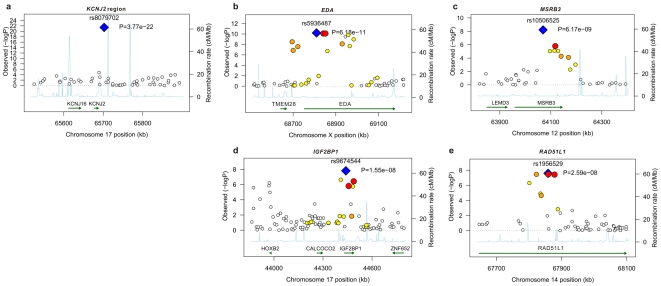
Linkage disequilibrium and association at loci reaching genome-wide significance for primary tooth development in meta-analysis of NFBC1966 and ALSPAC. (A) *KCNJ2* gene region for time to first tooth eruption. (B) *EDA* gene region for time to first tooth. (C) *MSRB3* gene region for time to first tooth. (D) *IGF2BP1* gene region for number of teeth at 12 months (SNP with high *P* at 44000 kb is that near *HOXB2*, rs6504340). Note: This is a gene-rich region, so most genes are omitted to simplify the plot. (E) *RAD51L1* gene region for number of teeth at 12 months. -log_10_ p-value is plotted against genomic position (NCBI build 36). Most significant SNP in each region is plotted in blue, r^2^ with top SNP is colour coded red (0.8 – 1.0), orange (0.5 – 0.8), yellow (0.2 – 0.5), and white <0.2. Gene annotations are based on Genome Browser (RefSeq Genes) and arrows represent direction of transcription. Recombination rate is estimated by LDhat using HapMap CEU sample. All r^2^ values are calculated in NFBC1966.

**Figure 2:**
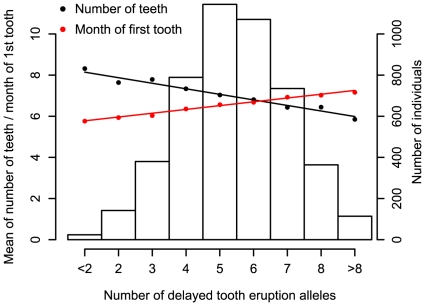
Meta-analysis for primary tooth development by genotype for the five SNPs attaining genome-wide significance. Estimates and 95% confidence intervals for regression coefficients are given for the effect of delayed teething allele in Gaussian regression on time to first tooth and an ordinal regression on number of teeth.

We tested 300,766 SNPs common to both studies (each used the Illumina platform). The analyses were adjusted for sex, gestational age and population structure (Materials and Methods). Results for the two cohorts were combined using fixed effects inverse variance meta-analysis. Five genetic loci were identified at genome-wide significance (*P*<5×10^−8^). Table 1 shows the top-ranking SNPs at each locus (see also Figure 1 and Figure 2, Figures S1, S2, S3). For all SNPs the allele associated with a delay in tooth eruption was associated with fewer teeth at the end of infancy. Table S1 shows details of the functions of genes linked to the identified loci.

**Table 1:** The top GWA signals at each locus from a meta-analysis of NFBC1966 and ALSPAC.

In (closest) gene/locus	SNP	Chromosome (Position)	Effect/Other allele	Frequency of effect allele NFBC ALSPAC		Time to first tooth eruption				Number of teeth				
						*P* value NFBC	*P* value ALSPAC	% var NFBC/ALSPAC	Overall *P*	*P* value NFBC		*P v*alue ALSPAC	% var NFBC/ALSPAC	Overall *P*
**SNPs at genome-wide significance in meta-analysis (** ***P*** **<5×10^−8^)**														
*(KCNJ2)*	rs8079702	17 (65,702,421)	G/A	0.391	0.424	1.89×10^−17^	1.62×10^−6^	1.62/1.48	**3.77×10^−22^**	7.78×10^−12^		3.22×10^−4^	1.15/1.05	**1.24×10^−14^**
*EDA*	rs4844096	X (68,722,043)	G/A	0.423	0.466	6.21×10^−6^	3.56×10^−4^	0.42/0.77	**2.61×10^−8^**	3.79×10^−9^		2.65×10^−3^	0.73/0.52	**4.57×10^−11^**
“	rs5936487	X (68,809,641)	G/A	0.390	0.462	6.65×10^−8^	6.90×10^−5^	0.50/0.99	**6.18×10^−11^**	3.89×10^−8^		2.10×10^−3^	0.52/0.57	**3.36×10^−10^**
*MSRB3*	rs10506525	12 (64,069,645)	C/T	0.266	0.375	1.29×10^−6^	5.80×10^−4^	0.46/0.71	**6.17×10^−9^**	2.56×10^−4^		5.60×10^−4^	0.18/0.74	8.67×10^−7^
*IGF2BP1*	rs9674544	17 (44,439,710)	G/A	0.462	0.484	2.97×10^−5^	5.44×10^−3^	0.25/0.40	8.33×10^−7^	2.29×10^−4^		9.86×10^−7^	0.27/1.61	**1.55×10^−8^**
*RAD51L1*	rs1956529	14 (67,858,677)	T/C	0.383	0.364	1.07×10^−3^	5.07×10^−4^	0.16/0.73	1.32×10^−5^	4.95×10^−6^		1.23×10^−3^	0.51/0.60	**2.59×10^−8^**
**SNPs with suggestive evidence (5×10^−6^>** ***P*** **>5×10^−8^) in meta-analysis**														
2q35	rs6435957	2 (217,586,454)	T/C	0.371	0.309	0.021	0.250	0.21/0.00	9.99×10^−3^	6.85×10^−6^	0.017		0.37/1.10	3.64×10^−7^
6q21	rs9386463	6 (106,200,750)	G/A	0.446	0.483	4.55×10^−6^	0.047	0.38/0.14	5.99×10^−7^	8.29×10^−3^	0.586		0.08/0.00	0.011
(*HOXB1, HOXB2*)	rs6504340	17 (43,972,018)	G/A	0.224	0.209	3.00×10^−3^	0.136	0.12/0.01	9.40×10^−4^	2.86×10^−5^	6.00×10^−3^		0.44/0.34	6.06×10^−7^
6q22	rs2817937	6 (121,140,156)	C/T	0.124	0.097	0.110	0.400	0.10/0.00	0.070	6.23×10^−6^	0.152		0.25/0.01	3.00×10^−6^
(*HMGA2*)	rs12424086	12 (64,650,776)	C/T	0.228	0.304	7.78×10^−5^	0.027	0.29/0.22	7.57×10^−6^	9.77×10^−5^	0.011		0.22/0.41	3.64×10^−6^

SNPs at genome wide significance *P*<5**×**10^−8^ and SNPS with suggestive evidence at 5**×**10^−6^>*P*>5**×**10^−8^. The *P* value of each cohort is corrected for sex, gestational age and population structure using principal components and genomic control. The combined *P* value is calculated using a fixed effects inverse variance meta-analysis. When no gene is within 50 kb of the SNP the chromosome band is given. Positions of SNPs are reported in NCBI build 36 coordinates. The alleles all refer to the forward strand. The effect allele is defined as the allele associated with later tooth eruption and a smaller number of teeth. % var is the percentage of variance explained by each SNP. *P* values attaining overall GWA significance are in bold.

The strongest association with both phenotypes was for SNP rs8079702, located 15 kb downstream of *KCNJ2* (inward rectifier potassium channel 2) (*P* = 3.77×10^−22^ for time of first tooth, *P* = 1.24×10^−14^ for number of teeth; Table 1). There are no SNPs in *KCNJ2* in our data, but rs8079702 had highest correlation with SNP rs4328485 which was the closest available SNP to *KCNJ2* (r^2^ = 0.17; 1 kb away). *KCNJ2* has been implicated in Pierre Robin sequence [7] and Andersen-Tawil syndrome [8], which show abnormalities in tooth development (missing teeth, delays in eruption) and are characterized by craniofacial anomalies such as narrowing of the jaw and cleft palate [8]. The second strongest association was for SNP rs5936487, located within the *EDA* (ectodermal dysplasia protein) gene (*P* = 6.18×10^−11^ for time of first tooth, *P* = 3.36×10^−10^ for number of teeth). *EDA* was fundamental in forming the first teeth in organisms [9], and mutations cause hypohidrotic ectodermal dysplasia (HED) and non-syndromic disorders of tooth agenesis [10].

The three remaining loci at genome-wide significance (*P*<5×10^−8^) have SNPs located within the genes *RAD51L1* (RAD51-like1), *IGF2BP1* (insulin-like growth factor 2 mRNA binding protein 1) and *MSRB3* (methionine sulfoxide reductase B3). *RAD51L1* is involved in DNA repair and a variant in the gene has been found to confer susceptibility to breast cancer [11]. It is responsible for protein kinase activity, and the injection of activators of protein kinase C (PKC) in rats causes delays in tooth eruption [12]. *IGF2BP1* regulates the growth factor *IGF2*, and knockouts of the gene in mice suggest a role in organ development [13], while its expression is associated with ovarian cancer [14]. A microarray study in the developing mouse molar tooth found *MSRB3* to be in the top 100 most expressed genes of 34,000 examined [15].

Each of the associated SNPs explain a small fraction of the residual phenotypic variation in time to first tooth (0.2%–1.6%, NFBC1966; 0.4%–1.5%, ALSPAC) and number of teeth by one year (0.2%–1.2%, NFBC1966; 0.5%–1.6%, ALSPAC), after controlling for sex and gestational age. Selecting the SNP with the most extreme signal for either phenotype to represent each locus (“top SNPs”), and analysing them together, the additive effects of these five top SNPs explain 2.9% of the variance of both tooth eruption time and number of teeth in the NFBC1966, and 4.2% and 4.0% of the variance in tooth eruption and number of teeth in ALSPAC. Without a suitable external replication cohort these estimates were derived in the two discovery cohorts and therefore may overestimate the true values due to the “winner's curse”. GWA studies have thus far explained only a small proportion of heritability [16], and our estimates are comparable with the variance explained in human height by a GWA study [17]. In order to identify variants with lower effect sizes or rarer variants larger sample sizes would be required.

**Figure 3:**
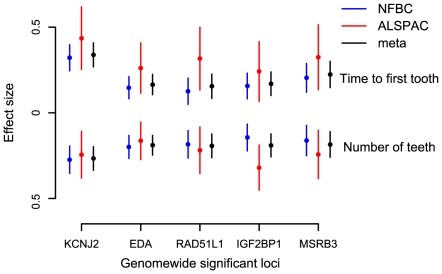
The combined impact of the delayed tooth eruption alleles in 5 identified loci at *P*<5×10^−8^ in the NFBC1966. Subjects are classified by the number of delayed tooth eruption alleles. SNPs are chosen so that they had the strongest signal for number of teeth at each locus. Mean time of first tooth eruption is plotted in red and number of teeth by the age of one year in black. The bars represent the number of individuals for each count of ‘delayed tooth eruption’ alleles. The line through points is a linear regression fit.

We also summarized the predictive power of the five top SNPs by defining a ‘delayed tooth eruption’ measure as the number of alleles across the SNPs that delay tooth eruption. Figure 3 shows the number of delayed tooth eruption alleles against the mean of both time to first tooth eruption and number of teeth by one year in NFBC1966. Individuals with 8 or more delayed eruption alleles (10% of NFBC1966) have an average of 1.5 fewer teeth at 12 months, and later tooth eruption by 1.1 months, compared to individuals with 3 or fewer such alleles (11% of NFBC1966). Figure S4 shows the same plot for time to first tooth in ALSPAC.

In addition to the five loci attaining genome wide significance, there were 5 loci with SNPs that had *P-*values between 5×10^−6^ and 5×10^−8^ (Table 1). We investigated the biological functions of nearby genes to see if any of these loci were related to tooth development. These signals included SNP rs6504340, which is located between the developmental regulatory genes *HOXB1* (homeobox B1) and *HOXB2* (homeobox B2). Although previous studies have indicated that tooth development is independent of a Hox patterning program [18], Homeobox genes have recently been shown to be expressed in the dental mesenchyme in the pharyngeal teeth of bony fishes [9]. SNP rs6504340 lies 500 kb upstream of rs9674544 in *IGF2BP1*, but the two SNPs show almost no linkage disequilibrium with each other (r^2^ = 0.002 in NFBC1966 and r^2^ = 0.006 in ALSPAC). Furthermore, a test for association of rs6504340 conditional on rs9674544 was significant (*P* = 6.3×10^−5^ in NFBC1966 and *P* = 0.01 in ALSPAC; Materials and Methods), indicating that these represent two independent signals (Figure 1). We also identified three SNPs at 2q35, the most significant of which had r^2^ = 0.48 with a variant associated with breast cancer [11],[19], and SNP rs12424086 located close to the *HMGA2* gene and 6 kb away from rs1042725, the SNP identified by a GWA study for adult and childhood height [20].

Given the influence of tooth development on dental occlusion, we hypothesized that genetic determinants of early tooth eruption may associate with dental occlusion later in life. We tested for associations between the SNP with the most extreme signal for either phenotype at each of the 10 identified loci and defects in occlusion requiring orthodontic treatment by the age of 31 years in the NFBC1966 (data not available in ALSPAC). A total of 611 individuals (13.5%) reported a defect in occlusion that had required orthodontic treatment. Of the 10 SNPs tested, SNP rs6504340 (*HOXB* gene cluster) gave a significant association, where each G allele (associated with delayed tooth eruption and lower number of teeth in infancy, Table 1), increased the odds of having an occlusal defect requiring orthodontic treatment by 35%, after adjusting for sex (odds ratio (OR) = 1.35, 95% CI = 1.16–1.57; *P* = 1.13×10^−4^; further adjustment for gestational age did not change the result). A smaller number of teeth at 1 year also predicted higher risk of orthodontic treatment (OR = 1.05, 95% CI = 1.01–1.09; *P* = 0.009). However, when number of teeth or time to first tooth were included in the model with dental occlusion as outcome, the associations with the G allele remained (*P* = 0.001, *P* = 1.71×10^−*4*^), suggesting an independent association between rs6504340 and dental occlusion.

## Discussion

Teeth and several other organs have common growth and developmental pathways during early life [21]. The genes at the loci identified in our study have roles in organogenesis, growth and developmental processes, and cancer. Mutations in three of the genes lead to altered organogenesis and development; *KCNJ2* (teeth, jaws, palates, ears, fingers, toes), *EDA* (teeth, hair, sweat glands, salivary glands) and *IGF2BP1* (intestines) [8],[13],[22]. Of the loci at suggestive levels of significance, the *HOXB* gene cluster is an established regulator of development, and the *HMGA2* gene has previously been associated with adult height [20]. Normal development and cancer both involve shifts between cell proliferation and differentiation [23] and genes regulating organ-specific growth are known to be involved in oncogenesis [24]. A previous study identified a common genetic link between an abnormal tooth development and cancer [25]. From our identified loci, *IGF2BP1* and *RAD51L1* have been implicated in cancer [11],[14] as have *HOXB2*, 2q35, and *HMGA2* [19],[26],[27].

We provide the first detailed insight into the genetic architecture of primary dentition and our findings could have implications for the study of other developmental and organogenic processes. Exploiting the availability of longitudinal cohort data [28] we found an association between a variant within the *HOXB* gene cluster and the requirement for orthodontic treatment due to defective occlusion by the age of 31 years. Further GWA studies of developmental processes during infancy may establish whether the genetic determinants of infant development can contribute to the study of chronic diseases, such as cancer, that occur later in life.

## Materials and Methods

### Study population and phenotype description

The data was derived from two genome-wide scans of the geographically defined prospective birth cohorts; the NFBC1966 and ALSPAC. The NFBC1966 followed pregnancies in the two northernmost provinces of Finland with expected delivery dates in 1966. ALSPAC recruited mothers during pregnancy with expected dates of delivery between April 1991 and December 1992 from Bristol and the surrounding area in the South West of England. A total of 4,564 samples were available from the NFBC1966 and 1,518 from ALSPAC. In both cohorts, two separate measures of primary tooth development were collected: i) date of first tooth eruption (in months), and ii) number of teeth (measured at 12 months in NFBC1966 and 15 months in ALSPAC). In the NFBC1966 date of first tooth eruption and number of teeth was gathered by public health professionals during children's monthly visits to child welfare centers (parents carried a booklet where they had recorded the developmental milestones reached). In ALSPAC, parents reported the date of first tooth eruption and number of teeth at 15 months on a questionnaire. In order to ascertain the accuracy of the parental responses, a subsample were examined and validated by a dentist. Information on date of first tooth eruption was available for 4,523 individuals in the NFBC1966 (99% of available GWA samples) and 1396 (92%) in ALSPAC and for number of teeth, 4,326 (95%) in the NFBC1966 and 1,426 (94%) in ALSPAC. All aspects of the study were reviewed and approved by the Ethics Committee of the University of Oulu and the ALSPAC Law and Ethics Committee and by the respective local research committees. Participants (in NFBC1966) and parents (in ALSPAC) gave written informed consent.

### Genotyping

The Illumina HumanCNV370-Duo DNA Analysis BeadChip was used for genotyping the NFBC1966, and Illumina HumanHap317K BeadChip for ALSPAC. The genotyping and quality control procedures have been described elsewhere [29],[30]. SNPs were excluded from the analysis if the call rate in the final sample was <95%, if there was a lack of Hardy-Weinberg Equilibrium (HWE) (*P*<10^−4^ in NFBC1966, *P*<5×10^−7^ in ALSPAC), or if the MAF was <1%. After quality control, 329,091 SNPs in NFBC1966 and 310,611 in ALSPAC were available. We report here the results from the 300,766 genotyped SNPs common to both studies.

### Statistical analyses

Age of first tooth eruption in the NFBC1966 was recorded in months, such that the first tooth could have erupted at any time between the end of previous month and the end of the recorded month. In ALSPAC it was recorded to the nearest month and 3 individuals were recorded as having no teeth after 15 months. To account for the censoring in the two cohorts the outcome was analyzed using parametric survival analysis in the R software package 2.7.1.The Gaussian distribution gave a good fit to the data in both cohorts and was used to model the underlying event time. Number of teeth in the NFBC1966 was recorded at 12 months. In ALSPAC, measurements were taken at around 15 months but there was variability in the exact time of measurement, therefore the ALSPAC analysis was adjusted for age of measurement. Teeth typically erupt in pairs from the upper and lower jaw (75% of children had an even number of teeth in the NFBC1966), making the Poisson distribution inappropriate for modeling the number of teeth. Therefore ordinal logistic regression was used as implemented by the polr function in the R package. Analyses of the X chromosome treated males as homozygous females. The allele frequencies of the identified SNPs on the X chromosome did not differ significantly between the sexes. GWA analyses were adjusted for sex, gestational age and population stratification using principal components (PC). Each analysis was corrected for population stratification separately by including those of the top 10 PCs that were associated with the phenotype at *P*<0.05 [31]. For number of teeth, PCs 3, 6 and 9 were included in ALSPAC and none in the NFBC1966. For time to first tooth eruption no PCs were included in ALSPAC and PC 2 was included in the NFBC1966. After correction by PCs, the estimated variance inflation factors [32] for date of first tooth eruption were 1.039 and 1.047 in ALSPAC and NFBC1966 respectively, and 1.011 and 1.039 for number of teeth. Genomic control [32] was then used to correct the residual population stratification. The variance inflation factors from the meta-analyses were 1.012 for number of teeth and 1.015 date of first tooth eruption.

Results from the two studies were combined using fixed effects inverse variance meta-analysis [33]. Analyses were performed using the statistical package R and metaMapper (a meta-analysis software developed in-house). Conditional analyses were calculated using the likelihood ratio test comparing ordinal regression models, one including rs9674544 and the other including rs9674544 and rs6504340. Variance explained by each SNP was computed as 1 minus the ratio of variance of residuals of the model with age, gestational age and SNP to variance of residuals of the model with just age and gestational age. To correct for overfitting, each individual's phenotype was estimated from a model that did not include that individual. The total variance explained by the five loci reaching genome-wide significance was calculated similarly using the most associated SNPs for each phenotype at each locus. Additional tests for association with orthodontic treatment used the SNPs most associated with number of teeth at the 10 loci at *P*<5×10^−6^. Table 1 reports the top GWA signals at each of the ten loci (i.e. the SNP with the strongest association with either time to first tooth eruption or number of teeth at age 1 year).

### URLs

Jackson Laboratory website, http://www.jax.org; NCBI, http://www.ncbi.nlm.nih.gov; R project, www.r-project.org; Uniprot, http://www.uniprot.org.

## Supporting Information

Figure S1 Manhattan plots for the 300,766 SNPs from the genome-wide association meta-analysis for (A) time to first tooth eruption, and (B) number of teeth at 12 months. The (blue) line indicates the genome-wide significance threshold (*P*<5×10^−8^).
(0.17 MB PDF)

Figure S2 Manhattan plots and linkage disequilibrium (LD) diagrams for five identified loci (*P*<5×10^−8^). (A) Locus 17q24 (*KCNJ2*), (B) Locus Xq13 (*EDA*), (C) Locus 14q24 (*RAD51L1*), (D) Locus 17q21.4 (*IGF2BP1*), and (E) Locus 12q14 (*MSRB3*). The (blue) line indicates the genome-wide significance threshold (*P*<5×10^−8^).
(0.89 MB PDF)

Figure S3 Quantile-quantile plots of observed -log_10_ *P* values versus the expectation under the null for (A) time to first tooth eruption and (B) number of teeth at 12 months. The most associated 10,000 SNPs from the meta-analysis are shown.
(0.07 MB PDF)

Figure S4 Additive effect of delayed tooth eruption alleles in identified loci in ALSPAC. Subject classified by the number of delayed tooth eruption alleles. SNPs chosen had the strongest signal for time to first tooth eruption at each locus. Mean time of first tooth eruption is plotted in black. The bars represent the number of individuals for each count of “delayed tooth eruption” alleles. Lines through points are linear regression fits.
(0.05 MB PDF)

Table S1 Summary of the candidate genes located within the top loci.
(0.08 MB PDF)
