# Correction: Genome-Wide Association Study Reveals Multiple Loci Associated with Primary Tooth Development during Infancy

**DOI:** 10.1371/annotation/e2a4252d-f903-4d2a-8acf-95d30a32a76c

**Published:** 2010-03-17

**Authors:** Demetris Pillas, Clive J. Hoggart, David M. Evans, Paul F. O'Reilly, Kirsi Sipilä, Raija Lähdesmäki, Iona Y. Millwood, Marika Kaakinen, Gopalakrishnan Netuveli, David Blane, Pimphen Charoen, Ulla Sovio, Anneli Pouta, Nelson Freimer, Anna-Liisa Hartikainen, Jaana Laitinen, Sarianna Vaara, Beate Glaser, Peter Crawford, Nicholas J. Timpson, Susan M. Ring, Guohong Deng, Weihua Zhang, Mark I. McCarthy, Panos Deloukas, Leena Peltonen, Paul Elliott, Lachlan J. M. Coin, George Davey Smith, Marjo-Riitta Jarvelin

The images for Figures 2 and 3 were mistakenly swapped. The titles and legends for both figures are correct, but the image for Figure 2 should be the image for Figure 3, and vice versa.

