# Supplementary figures and images for: Genome-Wide Association Study Reveals Multiple Loci Associated with Primary Tooth Development during Infancy
Source: PLoS Genet. 2010 Feb 26;6(2):e1000856. doi: 10.1371/journal.pgen.1000856 (PMC2829062; doi:10.1371/journal.pgen.1000856)

A

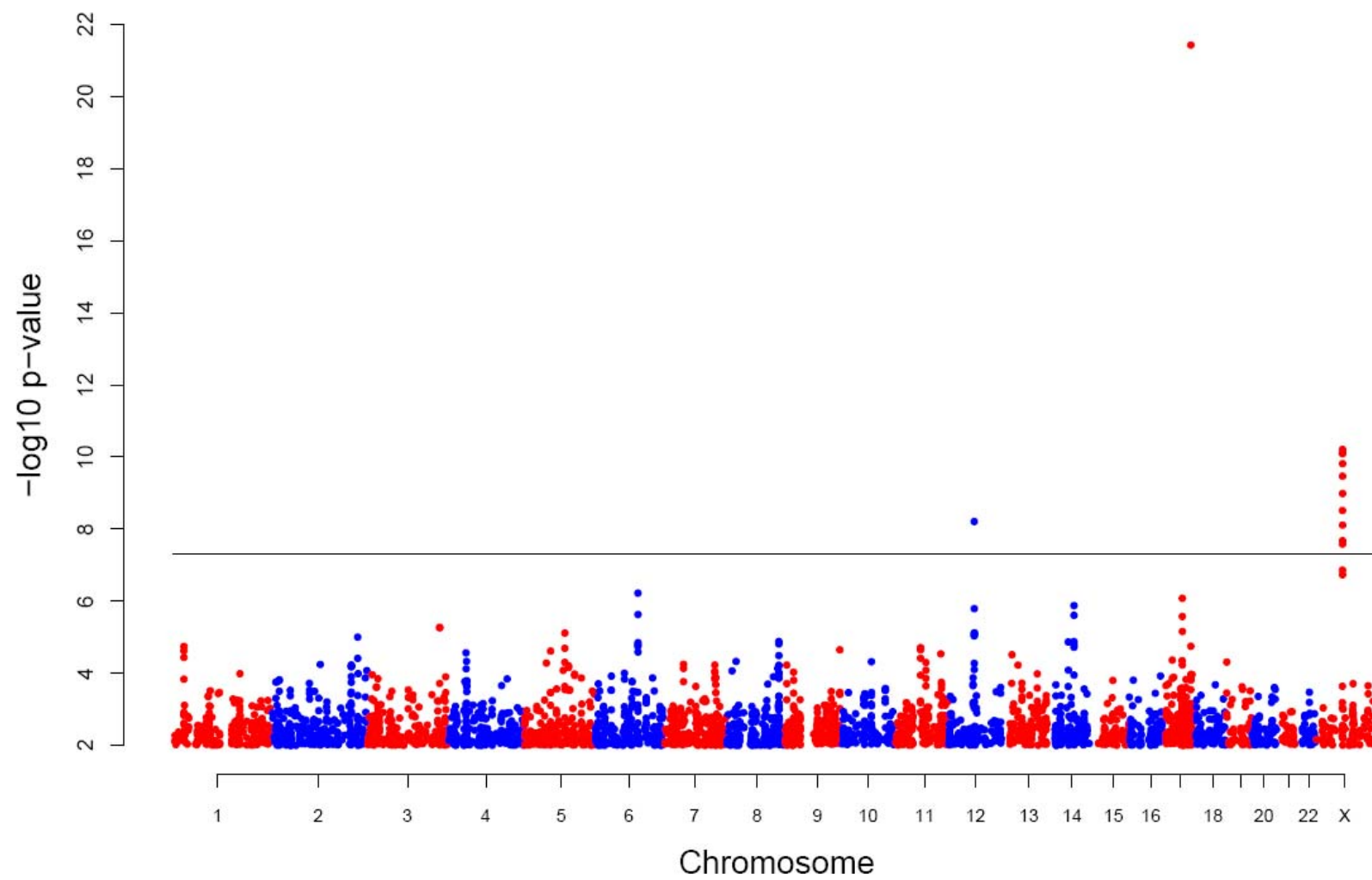

**B**

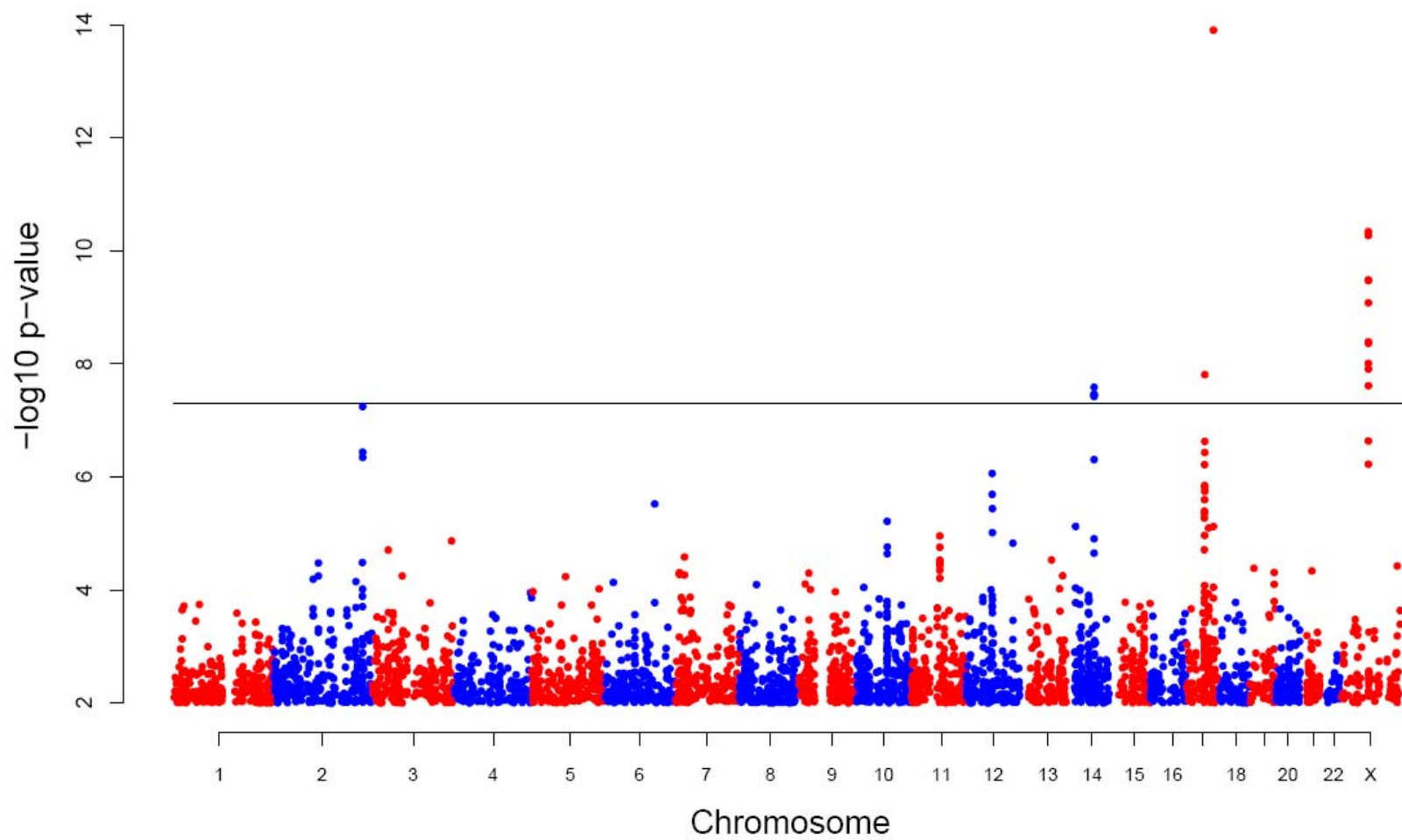

Supplement: Figure S1 — Manhattan plots for the 300,766 SNPs from the genome-wide association meta-analysis for (A) time to first tooth eruption, and (B) number of teeth at 12 months. The (blue) line indicates the genome-wide significance threshold (P<5×10^−8^). (0.17 MB PDF) [file pgen.1000856.s001.pdf]

**A**

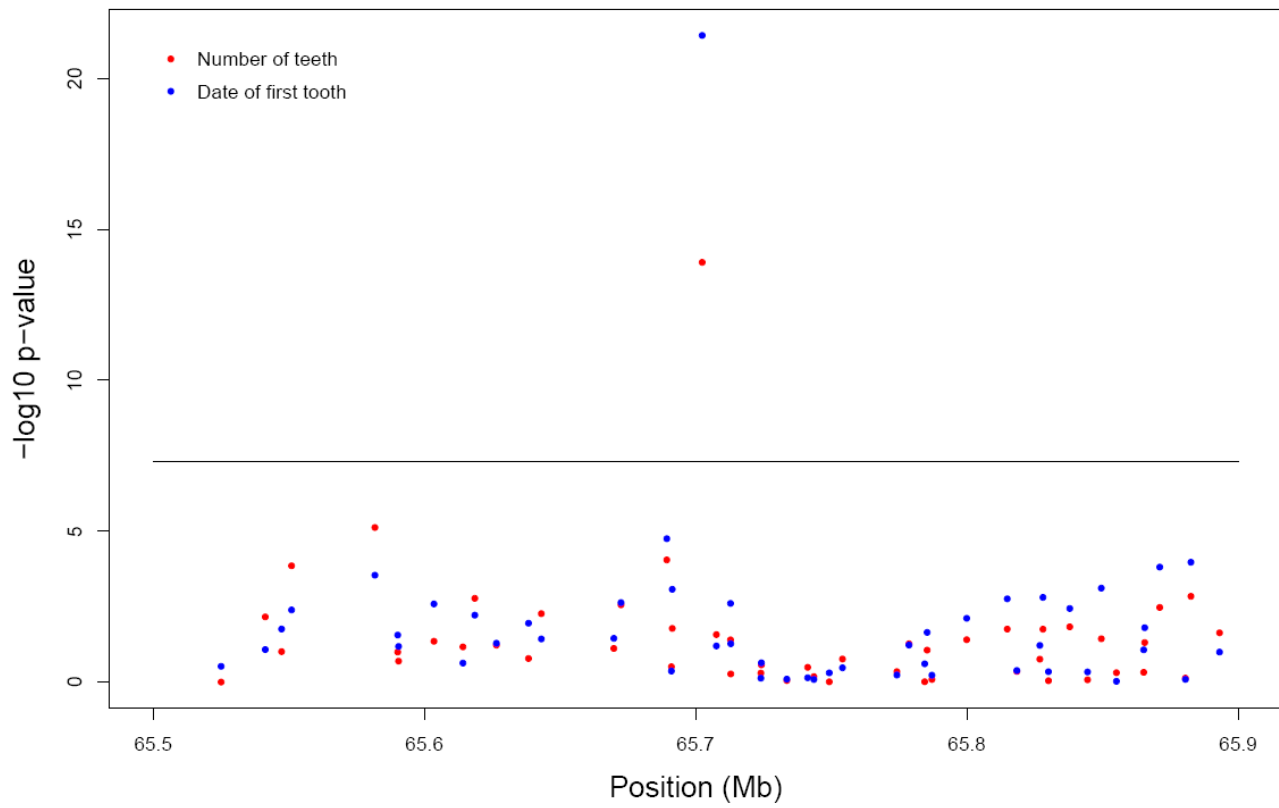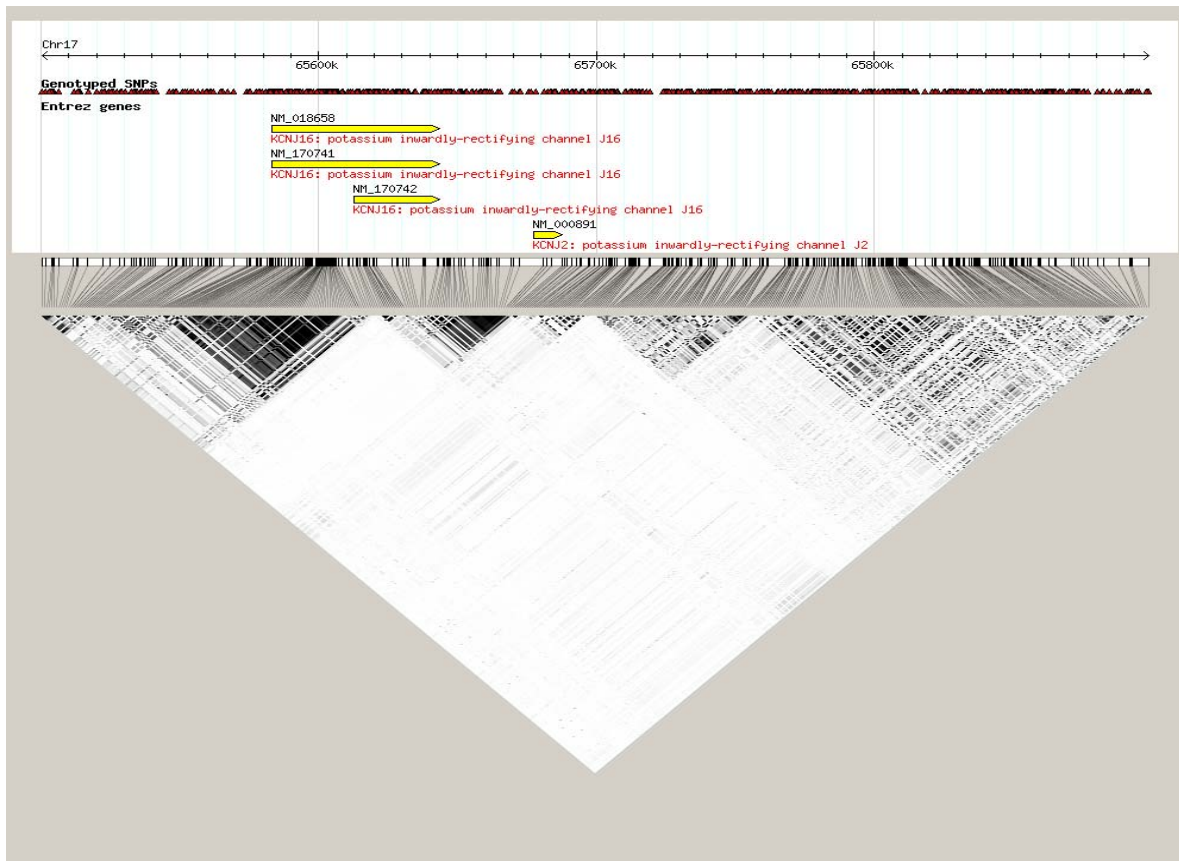

**B**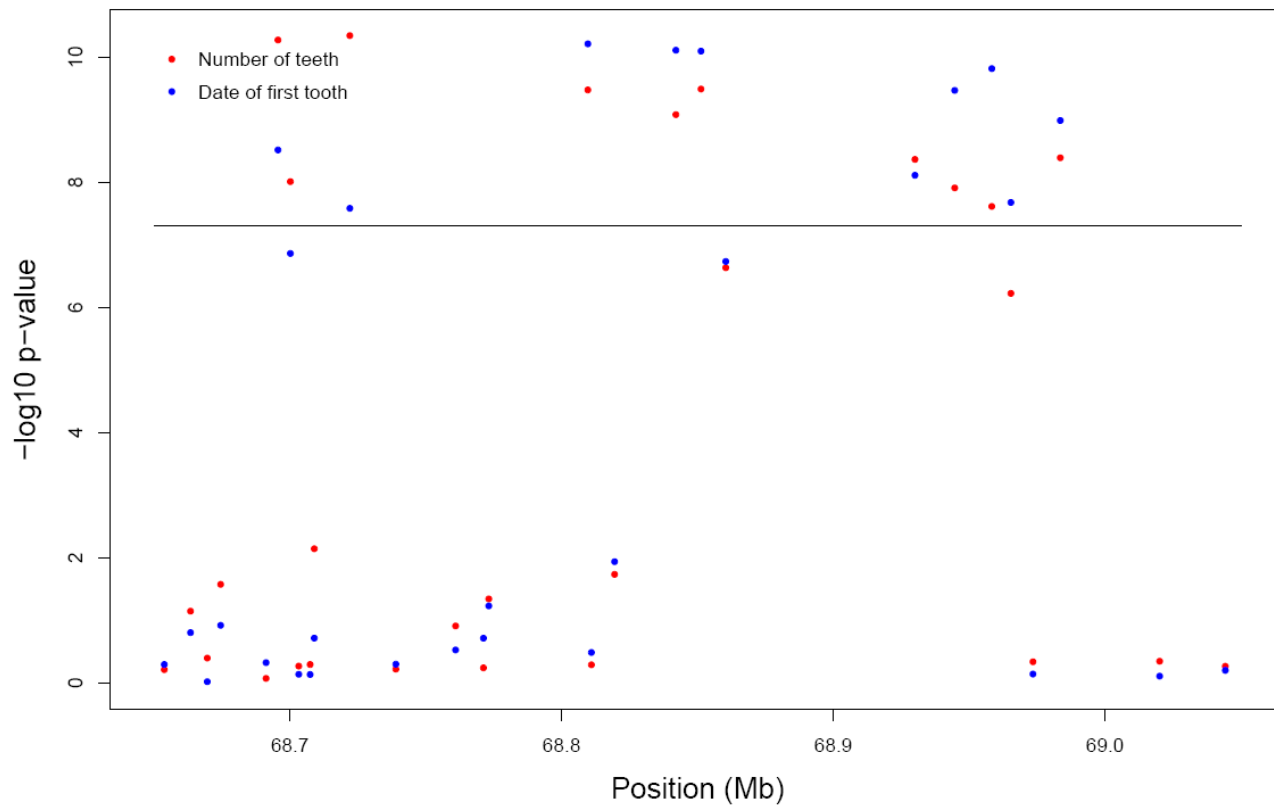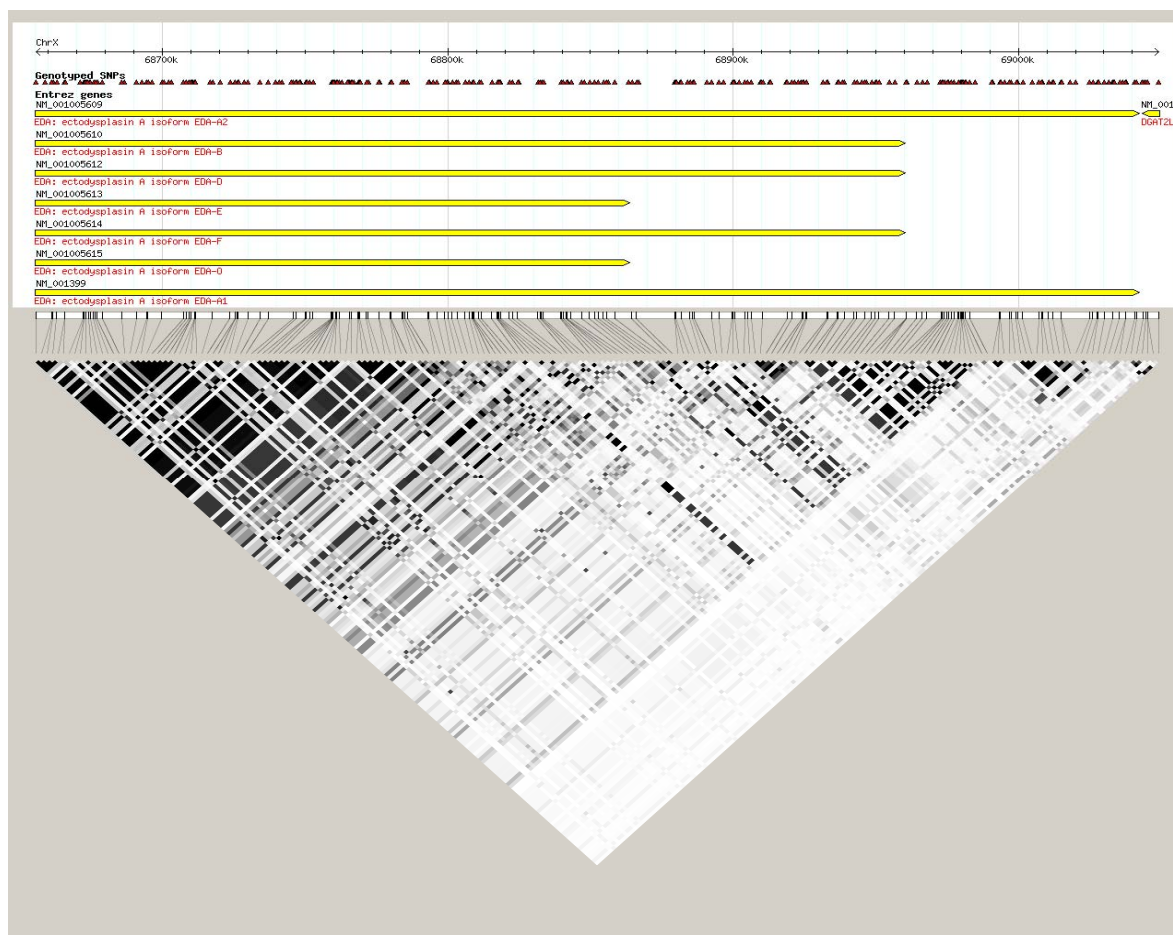

C

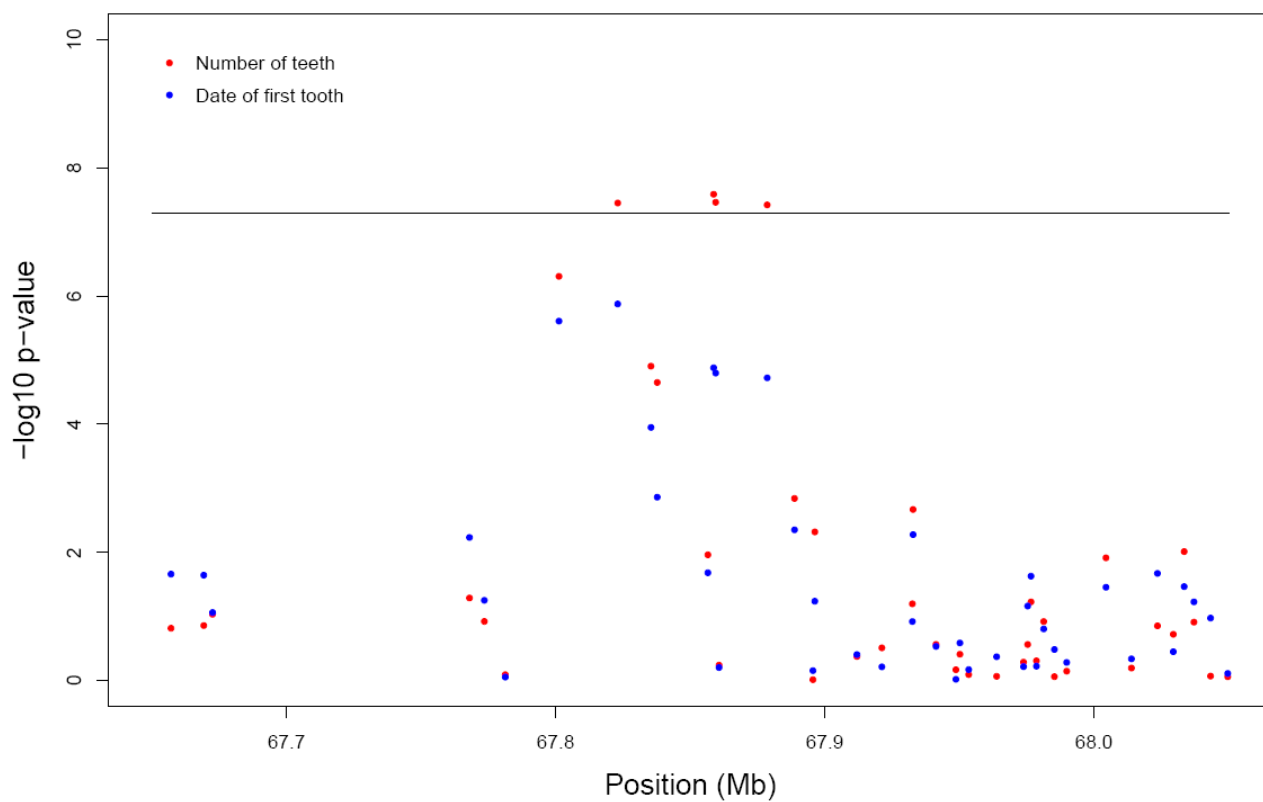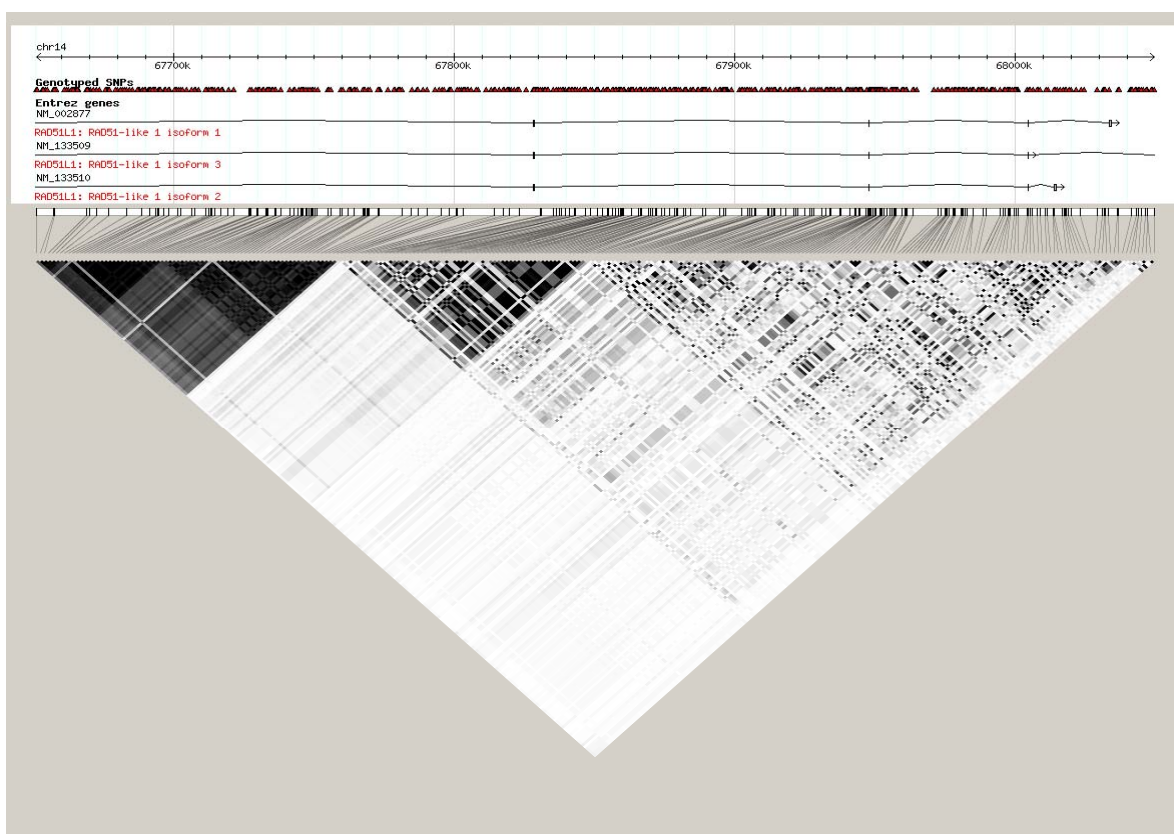

D

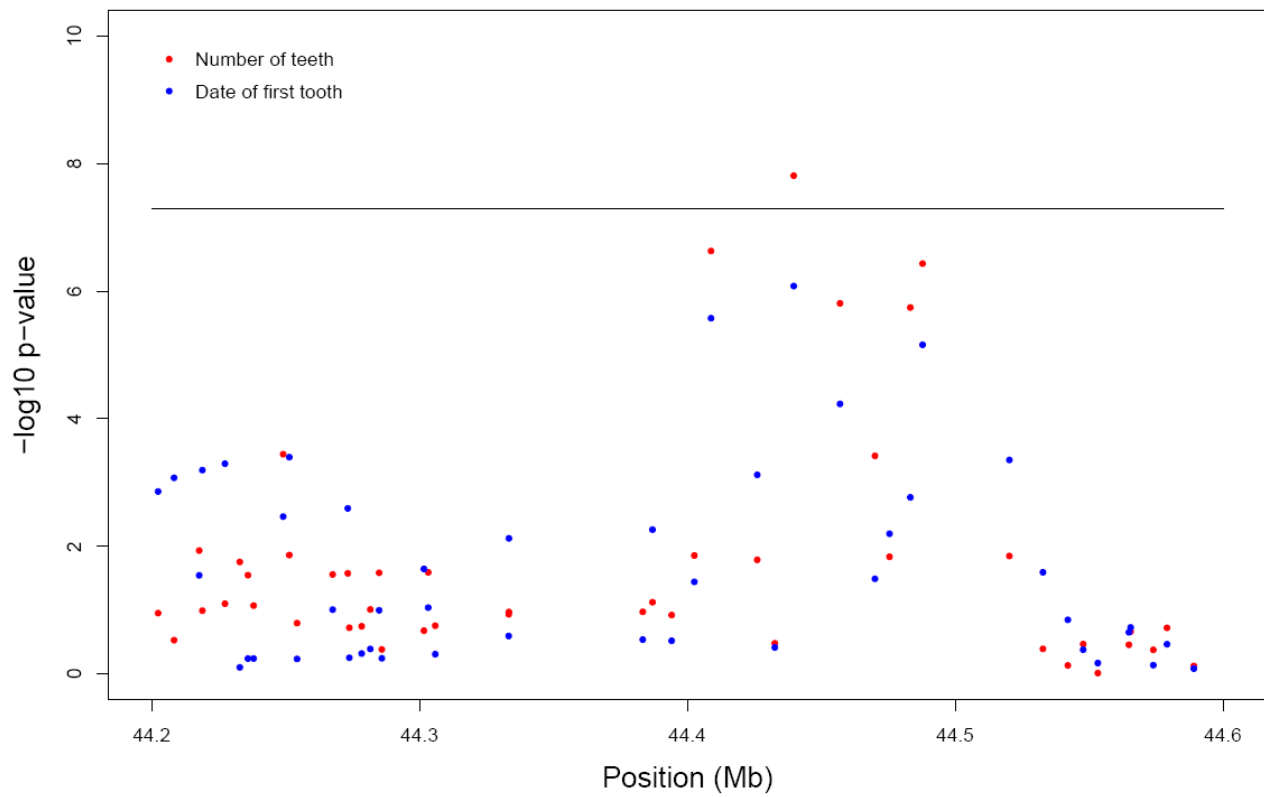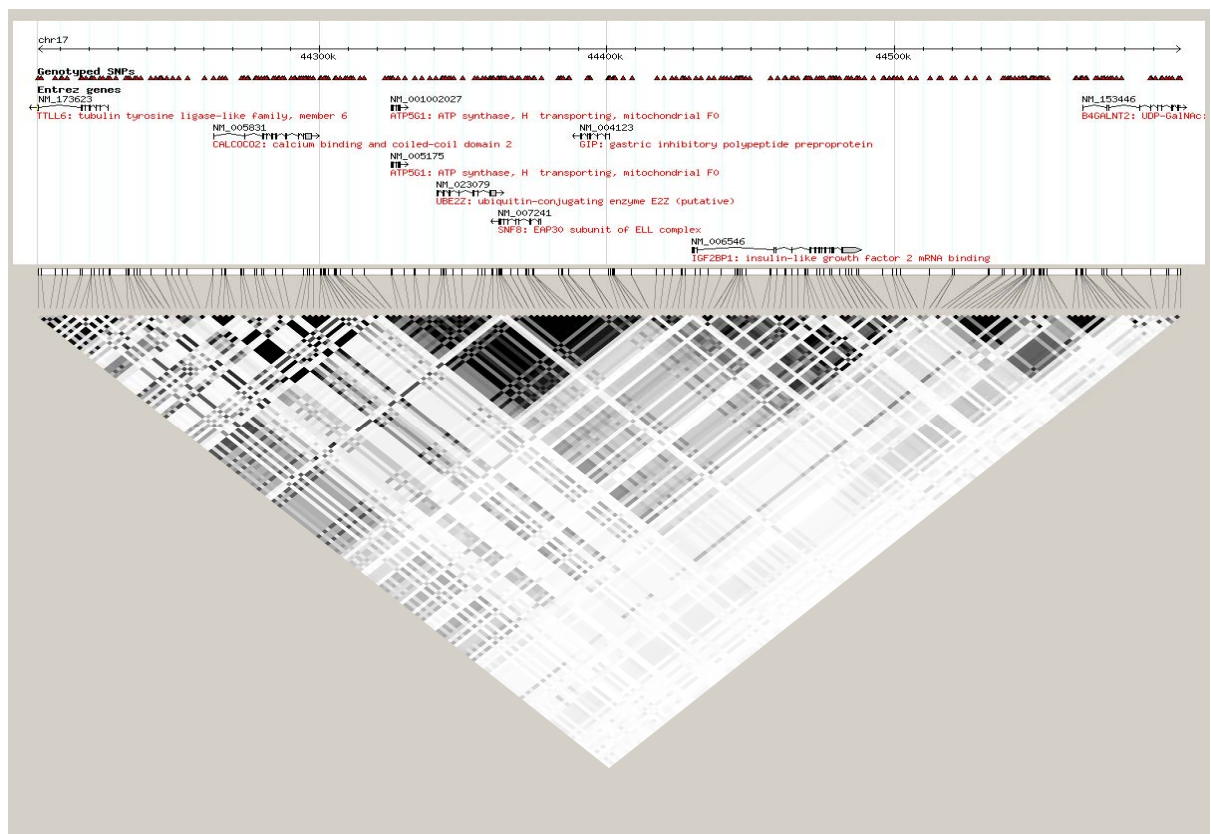

**E**

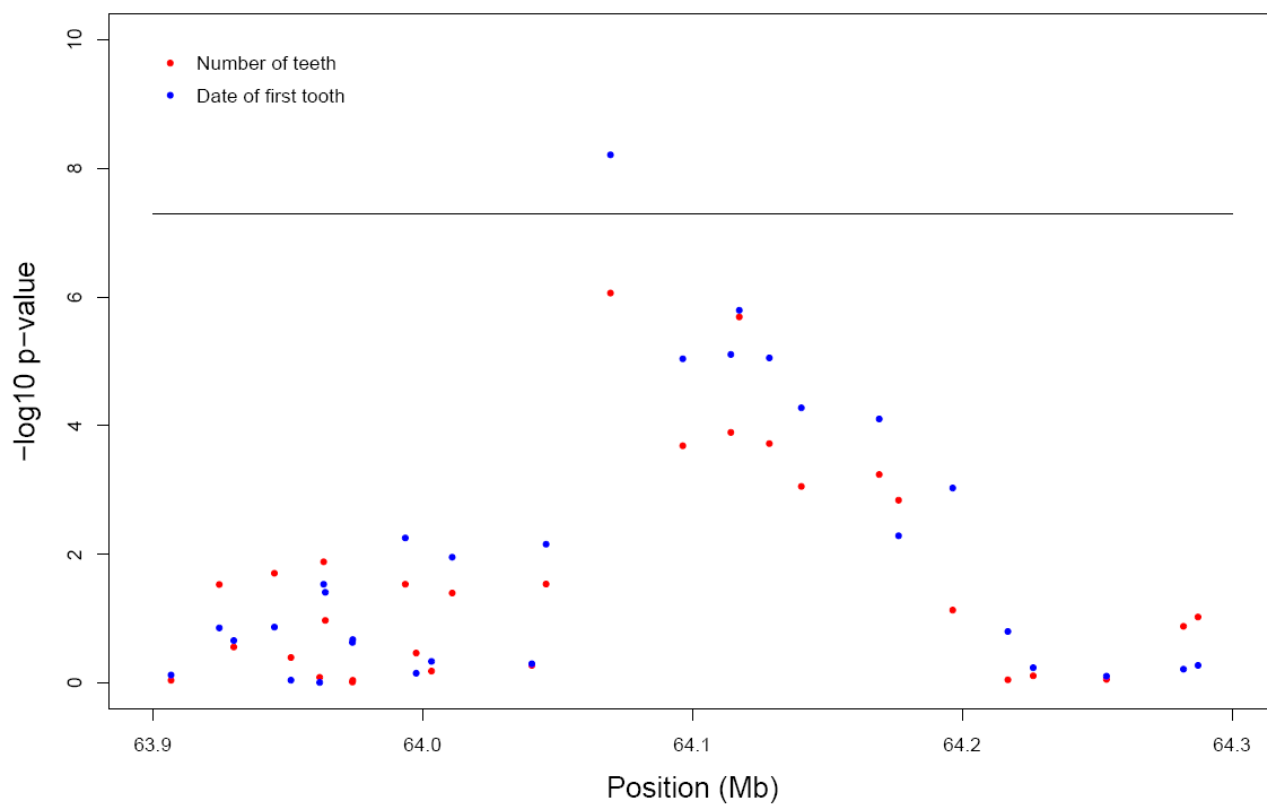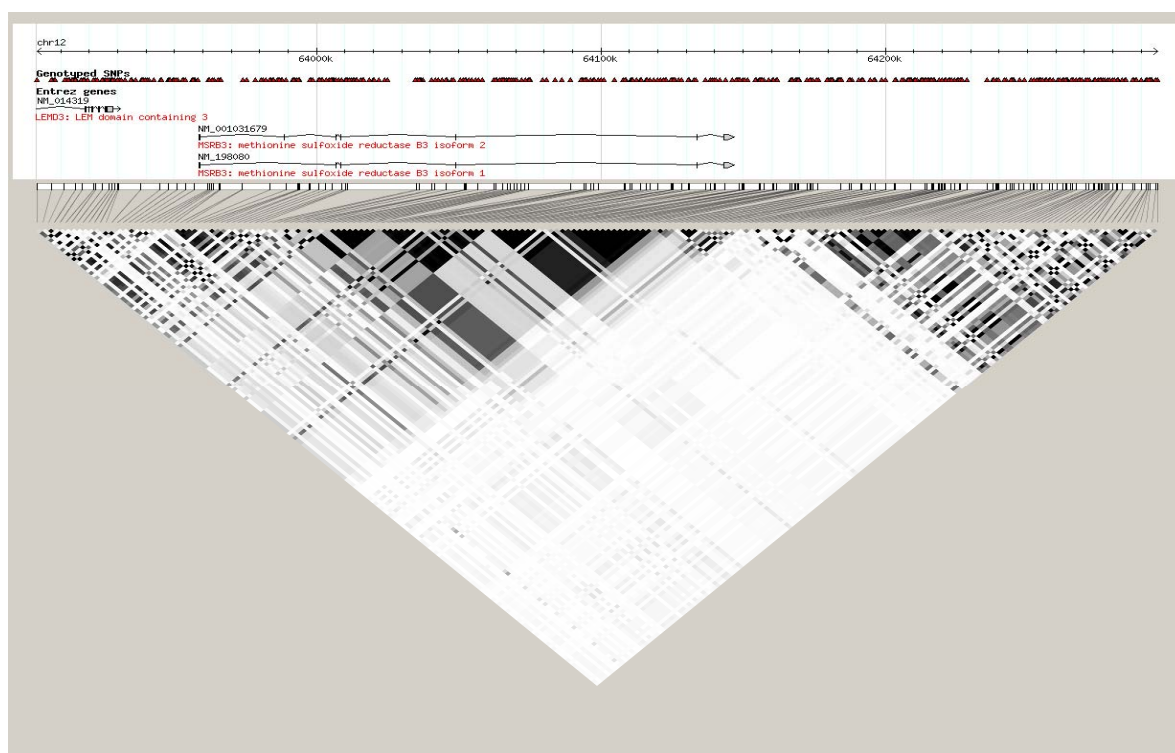

Supplement: Figure S2 — Manhattan plots and linkage disequilibrium (LD) diagrams for five identified loci (P<5×10^−8^). (A) Locus 17q24 (KCNJ2), (B) Locus Xq13 (EDA), (C) Locus 14q24 (RAD51L1), (D) Locus 17q21.4 (IGF2BP1), and (E) Locus 12q14 (MSRB3). The (blue) line indicates the genome-wide significance threshold (P<5×10^−8^). (0.89 MB PDF) [file pgen.1000856.s002.pdf]

A

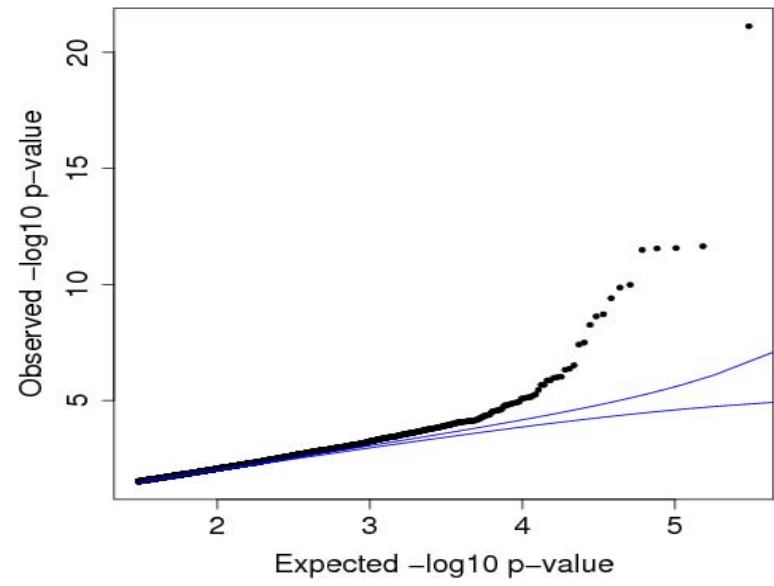

B

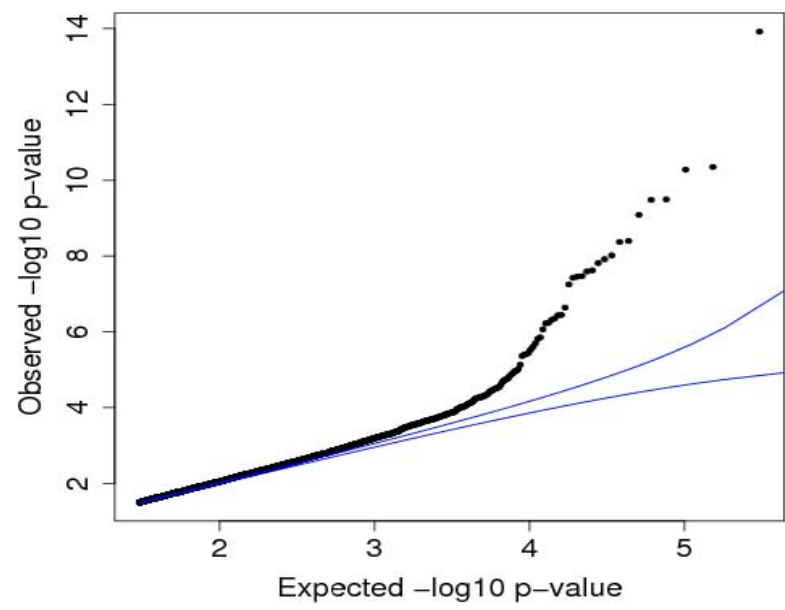

Supplement: Figure S3 — Quantile-quantile plots of observed -log10 P values versus the expectation under the null for (A) time to first tooth eruption and (B) number of teeth at 12 months. The most associated 10,000 SNPs from the meta-analysis are shown. (0.07 MB PDF) [file pgen.1000856.s003.pdf]

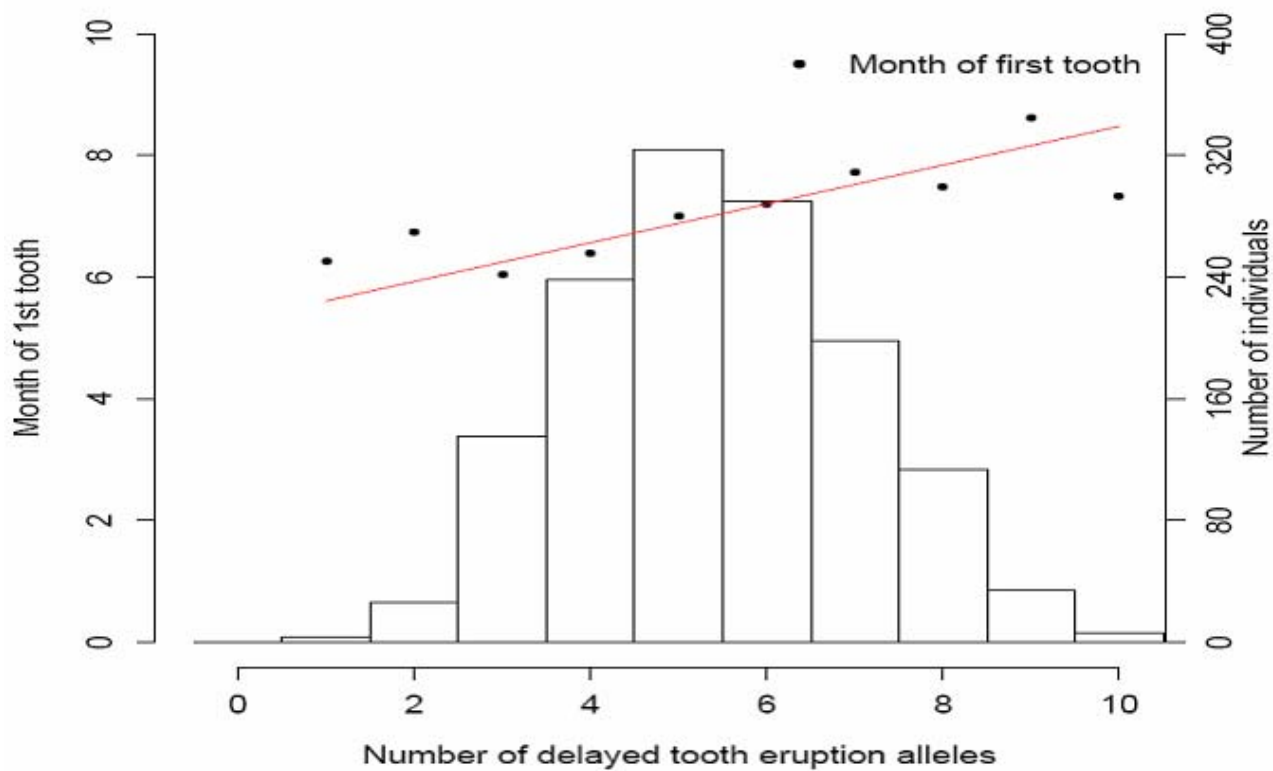

Supplement: Figure S4 — Additive effect of delayed tooth eruption alleles in identified loci in ALSPAC. Subject classified by the number of delayed tooth eruption alleles. SNPs chosen had the strongest signal for time to first tooth eruption at each locus. Mean time of first tooth eruption is plotted in black. The bars represent the number of individuals for each count of “delayed tooth eruption” alleles. Lines through points are linear regression fits. (0.05 MB PDF) [file pgen.1000856.s004.pdf]
