# Supplementary material for: Genome-Wide Association Study Reveals Multiple Loci Associated with Primary Tooth Development during Infancy
Source: PLoS Genet. 2010 Feb 26;6(2):e1000856. doi: 10.1371/journal.pgen.1000856 (PMC2829062; doi:10.1371/journal.pgen.1000856)
Supplement: Table S1 — Summary of the candidate genes located within the top loci. (0.08 MB PDF) [file pgen.1000856.s005.pdf]

**Table S1.** Summary of the candidate genes located within the top loci.

| Locus | Top SNPs[1)] | Candidate gene(s) [2)] | Syndromes caused by mutation in the gene | Knockout mouse phenotype/s | Details |
|---|---|---|---|---|---|
| 17q24 | rs8079702 | *KCNJ2* (inward rectifier potassium channel 2) | Andersen-Tawil syndrome (symptoms include craniofacial and skeletal anomalies, delay in tooth-eruption of permanent dentition, multiple missing teeth)<br>Pierre Robin sequence (symptoms include hypodontia and delayed tooth eruption) maps to this locus<br>Long-QT syndrome (associated with 10% of cases of Sudden Infant Death Syndrome, SIDS) | Complete cleft of secondary palate | The protein encoded by this gene, which has a greater tendency to allow potassium to flow into a cell rather than out of a cell, probably participates in establishing action potential waveform and excitability of neuronal and muscle tissues |
| Xq13 | rs4844096<br>rs5936487 | *EDA* (ectodermal dysplasia protein) | X-linked non-syndromic hypodontia – tooth agenesis (lack of primary or permanent teeth<br>X-linked hypohidrotic ectodermal dysplasia | Loss of teeth, hair, and sweat glands<br>Craniofacial and growth abnormalities | May be involved in cell-cell signaling during the development of ectodermal organs |
| 14q24 | rs1956529 | *RAD51L1*<br>(RAD51-like 1) | - | Early embryonic lethality<br>Severe growth retardation | Involved in DNA repair by homologous recombination<br>Mutations cause cell cycle G1 delay, cell apoptosis and severe failure of cell function |
| 17q21.44 | rs9674544 | *IGF2BP1* (insulin-like growth factor 2 mRNA binding protein 1) | - | Growth retardation<br>Impaired gut development<br>Increased neonatal lethality | Regulates IGF2 translation, may act as a regulator of mRNA transport to activated synapses in response to synaptic activity. Associated with the fragile X mental retardation protein |

| | | | | | |
|---|---|---|---|---|---|
| 12q14 | rs10506525 | *MSRB3* (methionine sulfoxide reductase B3) | - | - | Essential for catalytic activity. Increased expression in the developing mouse molar tooth |
| 17q21.43 | rs6504340 | *HOXB1*<br><br>(homeobox B1) | Homeobox genes are implicated in familial teeth agenesis | Excessive growth of the incisor teeth, which blocks the oral cavity and prevents feeding. Inability of newborns to feed could also reflect facial nerve defects, as this nerve innervates the muscles responsible for suckling<br><br>Facial motor nerve defects / facial paralysis<br><br>Early patterning defects in the hindbrain<br><br>Prenatal and perinatal lethality | A transcription factor with an important role in morphogenesis, including patterning calvarial bone during development. Involved in hindbrain segmentation and specification |
| | | *HOXB2*<br><br>(homeobox B2) | Homeobox genes are implicated in familial teeth agenesis | Facial paralysis with defects of the somatic motor component of the seventh facial nerve<br><br>Altered neurogenesis and neuronal differentiation patterns in the hindbrain | A nuclear protein which functions as a sequence-specific transcription factor involved in development<br><br>It regulates morphogenetic pathways that direct the regional identity and anteroposterior character of neural crest-derived structures like teeth |

| | | | | | |
|---|---|---|---|---|---|
| 12q14 | rs12424086 | *HMGA2* (high mobility AT-hook 2) | - | Shortened head<br><br>Decreased fetal size<br><br>Decreased body size<br><br>Decreased body weight<br><br>Increased resistance to diet-induced obesity | Functions as a transcriptional and growth regulating factor and is expressed predominantly during embryogenesis. Involved in mesenchymal differentiation and multicellular organismal development<br><br>Associated with height variability and cancerogenesis |

1) The SNP within the loci with the strongest association with either time to first tooth eruption or number of teeth at age 1 year.

2) A candidate gene is listed when, within the 50-kb window around the SNP, monogenic human and/or mouse phenotypes and/or expression results clearly suggest a plausible candidate (in this case, in 7 of the 10 identified loci). Information on each gene was obtained from the Uniprot, NCBI, and Jackson Laboratory website (Jackson Laboratory website, http://www.jax.org; NCBI, http://www.ncbi.nlm.nih.gov; Uniprot, http://www.uniprot.org).
